# When and How Workplace Helping Promotes Deviance? An Actor-Centric Perspective

**DOI:** 10.3389/fpsyg.2021.795610

**Published:** 2022-01-05

**Authors:** Hao Zhang, Chunpei Lin, Xiumei Lai, Xiayi Liu

**Affiliations:** ^1^School of Business Administration, Huaqiao University, Quanzhou, China; ^2^Business Management Research Center, Huaqiao University, Quanzhou, China

**Keywords:** caring helping, coaching helping, substituting helping, workplace deviance, emotional exhaustion, extrinsic career goals

## Abstract

Despite the vast academic interest in workplace helping, little is known about the impact of different types of helping behaviors on physiological and behavioral ramifications of helpers. By taking the actor-centric perspective, this study attempts to investigate the differential impacts of three kinds of helping behaviors (caring, coaching, and substituting helping) on helpers themselves from the theory of resource conservation. To test our model, 512 Chinese employees were surveyed, utilizing a three-wave time-lagged design, and we found that caring and coaching helping were negatively associated with workplace deviance, whereas substituting helping was positively associated with subsequent workplace deviance. Emotional exhaustion mediated the effects of three helping behaviors on subsequent workplace deviance. Moreover, employees' extrinsic career goals influenced the strength of the relationship between three helping behaviors and emotional exhaustion and the indirect effects of three helping behaviors on subsequent workplace deviance *via* emotional exhaustion. We discuss the implications of our findings for both theories and practices.

## Introduction

In highly turbulent environments, companies have increasingly relied on team-based work, thus increasingly encouraging employees' helping behaviors, which are inherently moral and virtuous in the workplace (Deckop et al., 2003). By taking a social-exchange perspective, scholars have long focused on the “bright side” of workplace helping, defined as “an affiliative and supportive behavior that results in the helper receiving benefits from others at work” (Harari et al., 2021). However, from the perspective of resource conservation, helpers often have different psychological and behavioral responses after their helping behaviors due to the limited resources (Lin et al., 2020). This difference also leads to contradictory conclusions in the existing research on the relationship between helping behavior and its subsequent behavior. Workplace deviance, as one of potential subsequent behaviors of workplace helping (Yam et al., 2017), refers to the voluntary behavior that damages an organization and/or its members by violating important norms in the workplace (Robinson and Bennett, 1995; Bennett and Robinson, 2000). Some employees appreciate that they acquire resources when helping others in the workplace. Workplace helping can trigger positive emotional states of helpers (e.g., authentic pride), which reduce the likelihood of deviant behaviors (Kim et al., 2018). However, some employees hold the view that helping behavior will deplete their own resources because it is not in their backyards (Koopman et al., 2020). They are prone to take a sense of psychological entitlement to transgress for granted, which makes them feel more comfortable to deviate after helping colleagues (Yam et al., 2017). Some scholars point out that the above contradictory views may be related to the multidimensional structure of helping behavior (Bamberger et al., 2017; Duan et al., 2019). Studies have shown that different types of helping behaviors have completely different effects on the psychology and subsequent behaviors of helpers (Shah et al., 2018; Lee et al., 2019). Therefore, it remains to be further explored as to how and when the different dimensions of helping behaviors will lead to deviating behaviors.

Building on the existing work, we will start from the process of individual resource gain and loss, and draw from conservation of resources theory to examine how different types of workplaces helping may motivate individuals to direct workplace deviant behavior. We address these problems by classifying workplace helping into *caring helping* (i.e., helping colleagues overcome negative emotions; Lee and Allen, 2002), *coaching helping* (i.e., sharing knowledge; Podsakoff et al., 1997), and substituting helping (i.e., substitute colleagues to complete work; McDonald et al., 2018) based on the extent to which a helper engages in helping coworkers. Helping behavior has both the characteristics of resource gain and resource depletion. When a certain type of workplace helping can obtain valuable results that meet personal goals, the helper's perception and emotion toward helping behavior in the workplace will become positive; meanwhile, helping behavior exhibits a resource-enhancing effect. Conversely, when helping colleagues get results that deviate from personal goals and are worthless, the helper's perception and emotions toward helping behavior in the workplace will become negative, and, at this time, helping behavior exhibits a resource depletion effect (Bamberger et al., 2017). The resource depletion effect will worsen the resource condition of the helper, leading to resource depletion, and leaving employees in a desperate situation of resources. Conservation of resources theory states that employees in desperate situations will trigger self-defense mechanisms to obtain resources by implementing irrational and aggressive behaviors, harming the interests of the organization and colleagues (Hobfoll et al., 2018). However, which helping behaviors will produce the effect of resource depletion, and which helping behaviors will produce the effects of resource enrichment, the current research literature does not give a clear response to the above questions. As mentioned above, it is highly likely that different workplace helping behaviors lead to different resource outcomes.

**Figure 1:**
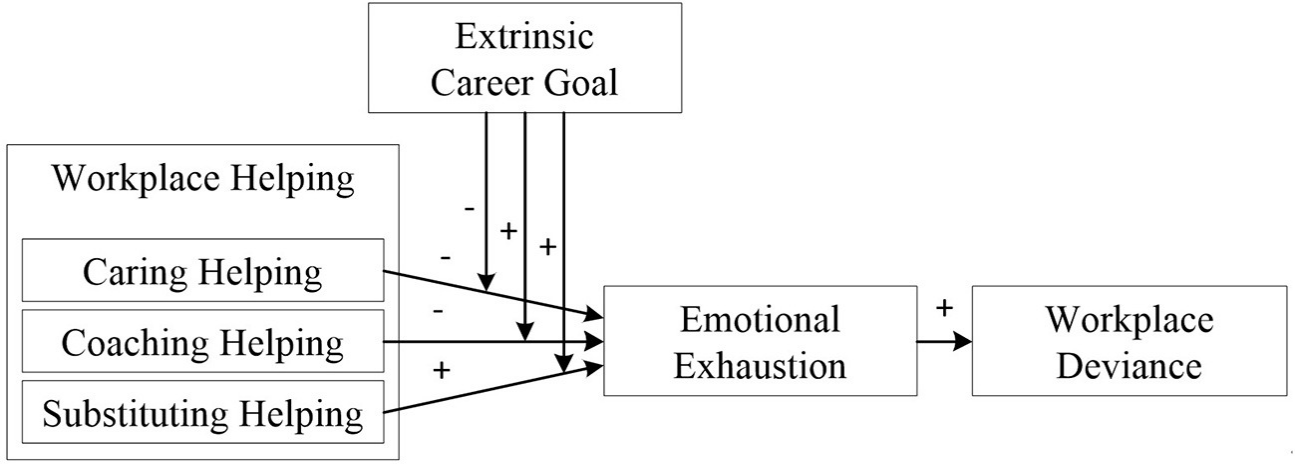
The conceptual mode of this research.

To model the relationship between different styles of workplace helping and subsequent deviance, we adopt a resource-based framework. Specifically, we draw upon conservation of resources theory to propose that caring and coaching helping is less *emotional exhausting* (i.e., feeling of “psychological resource availability”; Lin et al., 2020) to helpers than substituting helping. Unlike caring and coaching helping, helpers engaged in substituting helping are more emotionally exhausted because they spend more time and effort. We posit that this increased emotional exhaustion, in turn, triggers workplace deviance in resource-exhausted circumstances. Furthermore, we argue that the effects of workplace helping will depend on the helpers' *extrinsic career goals* (i.e., an individual's career goals including pursuing short-term extrinsic work outcomes such as salary; Seibert et al., 2013). Helpers with high extrinsic career goals will be more emotionally exhausted because the resource depletion by workplace helping prevents them from extrinsically motivating attributes such as financial rewards (Figure 1 illustrates our concept model).

The current research contributes to the existing literature in three ways. First, this research is to examine the relationship between different types of workplaces helping and deviance. Previous studies usually regard helping behavior as a whole to explore the relationship between helping behavior and deviant behavior, and draw different or even opposite conclusions (Yam et al., 2017; Koopman et al., 2020). We introduce conservation of resources theory as a theoretical lens that explains how different helping behaviors may lead to different consequences on deviant behavior. Secondly, we contend that emotional exhaustion plays an essential mediating role and in the process of workplace helping behavior to deviance behavior. Emotional exhaustion reflects prolonged physical, affective, and cognitive strain at work (Koopman et al., 2016). Existing research finds a positive relationship between organizational citizenship behavior and emotional exhaustion (Halbesleben and Wheeler, 2011). Helping behavior is a common type of organizational citizenship behavior, but its effect on emotional exhaustion has not been fully explored. In addition, research suggests that emotional exhaustion coexists with feelings of lack of energy, fatigue, anxiety, and frustration (Eissa and Lester, 2018); this is similar to the characteristics of deviant behavior. Third, we introduce extrinsic career goals as a moderating variable to clarify the important boundary effect of different types of helping behaviors. Previous study argues that employees are likely to have varying degrees of extrinsic career goals (Seibert et al., 2013). Extrinsic career goals play an important part in value judgment that affects employees' investment in resources; few studies consider their connection with organizational citizenship behavior and emotions.

## Theoretical Background and Hypotheses

### Workplace Helping and Workplace Deviance

Workplace helping includes caring helping, coaching helping, and substituting helping. Caring helping means that the helper does not directly intervene in the tasks related to the work but invests emotional resources to care for the recipient, such as helping colleagues overcome negative emotions (Lee and Allen, 2002), listen carefully to what your colleagues are saying (Williams and Anderson, 1991; Settoon and Mossholder, 2002), and so on. Coaching helping refers to the way that the helper helps others to improve their knowledge and work skills by investing cognitive resources to impart knowledge to the recipients and to provide advice and suggestions, which mainly include the sharing of professional knowledge (Podsakoff et al., 1997), sharing innovative ideas (Van Dyne et al., 1994), and so on. Substituting helping means that the helper directly intervenes in the work related to the recipient's help-seeking matter as a substitute by devoting all resources and fully or partially assumes additional work responsibilities, including sharing heavy tasks (Farh et al., 1997), substitute colleagues to complete work (McDonald et al., 2018), etc. Different types of helping behaviors input different resources, and the mechanisms for obtaining resource returns are also different.

Workplace deviance has been labeled as counterproductive behavior, antisocial behavior, or deviant workplace behavior, which affects almost all organizations (Robinson and Bennett, 1995; Robinson and O'Leary-Kelly, 1998). The antecedents of deviant behavior mainly include individual-level factors and situation-specific or organizational factors (Nair and Bhatnagar, 2011). Employees who deviate in the workplace are labeled as uncomfortable, irresponsible, or emotionally unstable (Mount et al., 2006; Berry et al., 2007), while those who help colleagues in the workplace are often considered as pro-social, pleasant, or conscientious in the organization (Organ, 1988; Lin et al., 2020). Previous studies have argued that these contradictory personal traits are difficult to concentrate on the same individual (Berry et al., 2007). Therefore, the behaviors of “good citizens” that damage the organization and colleagues are mainly affected by situational factors. Job stressors (such as helping pressure) are considered to be one of the situational inducements for deviant behaviors in the workplace (Meier and Spector, 2013; Koopman et al., 2020). Take helping pressure as an example. When supervisors actively encourage subordinates to help colleagues, or when subordinates feel that they should actively respond to colleagues' requests for help, subordinates will feel pressure to help, which will adversely affect their own cognition and emotions. At the cognitive level, helping colleagues will put pressure on helpers, making individuals inclined to be more morally disengaged (He et al., 2019), and causing employees to be unable to effectively restrain their own behavior; at the emotional level, individuals under heavy pressure will have more negative emotions and are more likely to vent their dissatisfaction by harming the interests of the organization or colleagues (Koopman et al., 2020). In addition, helping pressure can also allow employees to gain a sense of psychological entitlement so that the implementation of deviant behaviors can obtain permission on the level of moral perception (Yam et al., 2017).

Whether it is failures in self-regulation, venting ones anger, moral entitlement or disengagement, the reason why a helper becomes a perpetrator can be attributed to a sense of help pressure. Conservation of resources theory provides a more comprehensive and complete explanation framework for the influence mechanism of workplace helping behavior on deviant behavior. First of all, conservation of resources theory supports the idea that resources are perceived as anything that contribute to the achievement of individual goals (Halbesleben et al., 2014). Preserving, protecting, and obtaining resources are the main strategies for individuals to cope with stress (Hobfoll, 1989); both potential and actual loss of resources will cause individual tension and pressure (Hobfoll, 1989; Hobfoll et al., 2018). Secondly, workplace helping behavior is a double-edged sword for helpers, which can both eliminate and generate resources (Bamberger et al., 2017; Lin et al., 2017). Because of this, many inconsistent conclusions have emerged in the process of studying the impact of helping behaviors in the workplace. Scholars speculate that this is related to the multidimensional structural characteristics of helping behaviors in the workplace (Bamberger et al., 2017; Shah et al., 2018; Duan et al., 2019). Some types of helping behaviors (such as concerning and compassionating for colleagues) will consume fewer resources, while other types of helping behaviors (such as replacing absent colleagues to complete corresponding tasks) require the helper to devote all physical, cognitive, and emotional resources. Finally, the helping behavior that consumes more resources will become a source of stress and cause the resource exhaustion of the helper. In order to get rid of the resource dilemma, in the absence of external resource support, individuals are forced to activate the self-defense mode and supplement resources through improper means, thereby harming the interests of the organization and colleagues (Hobfoll et al., 2018).

According to the work engagement theory, the resources that employees can invest in their work include physical, cognitive, and emotional resources (Kahn, 1990). On the basis of previous research (Williams and Anderson, 1991; Van Dyne et al., 1994; Farh et al., 1997; Podsakoff et al., 1997; Lee and Allen, 2002; Settoon and Mossholder, 2002; McDonald et al., 2018), we explore the influence of three different types of helping behaviors on deviant behaviors from the perspective of helpers.

In terms of resource loss, the three types of helping behaviors require the helper to invest resources, but there are differences in the amount of resource loss. By analyzing the resource competition between the three types of helping behaviors and jobs, the differences can be better understood. Compared with caring and coaching helping, substituting helping will squeeze the time and energy of the helper and have the greatest impact on their own work (Bergeron, 2007). In terms of resource return, all three types of helping behaviors can gain resources, but there are significant differences in the cycle of resource gains. Caring and coaching helping occurs in work situations where the helper and the recipient are present at the same time, which contributes the helper to receive positive feedback and gratitude from the recipient in a shorter period (Lee et al., 2019). In sharp contrast, substituting helping occurs when the recipient is absent, which is not conducive to the resource gain spirals (McDonald et al., 2018). From the perspective of comprehensive resource loss and gain, caring and coaching helping is more likely to form a net increase in resources and stimulate a spiral of resource enhancement for the helper, while substituting helping is more likely to cause a net loss of resources and stimulate the helper's resource depletion (Hobfoll et al., 2018). Therefore, unlike caring and coaching helping, substituting helping will cause pressure on the helper, worsen the resource status, and more easily stimulate the helper's self-defense mechanism, leading to deviant behavior (Hobfoll et al., 2018). We thus hypothesize the following:

H1a: Caring helping is negatively related to workplace deviance.

H1b: Coaching helping is negatively related to workplace deviance.

H1c: Substituting helping is positively related to workplace deviance.

### The Mediating Role of Emotional Exhaustion

Emotional exhaustion is a manifestation of psychological stress that exhausts emotions and psychological resources (Verhoeven et al., 2003), which can be used to measure the availability of psychological resources (Lin et al., 2020). Emotional exhaustion is caused by persistent high work demands and workplace troubles (Eissa and Lester, 2018). Conservation of resources theory provides a theoretical explanation for whether and under what circumstances workplace helping behavior will lead to emotional exhaustion of the helper. According to the viewpoint of conservation of resources theory, when a kind of helping behavior causes a net loss of individual resources, the resource loss spiral will accelerate the loss of resources, causing the helpers to fall into resource dilemma, and the lack of resources will eventually lead to emotional exhaustion (Hobfoll et al., 2018). Caring and coaching help requires a lower amount of resources and has less resource conflict with their own work (Bergeron, 2007); on the other hand, substituting help requires the helper to share the workload of other colleagues and spare no effort solve the problems of other colleagues and affect the progress of the job (Koopman et al., 2016). Continuous substitution has helped to increase the job needs of helpers, causing trouble to their jobs, and being more prone to emotional exhaustion (Eissa and Lester, 2018).

Here, we believe that emotionally exhausted helpers will increase their deviant behaviors because they lack sufficient physical, cognitive, and emotional resources, accompanied by feelings of insufficient energy, fatigue, anxiety, and depression (Eissa and Lester, 2018), making it difficult for them to manage interpersonal relationships and conflicts of work resources (Jahanzeb and Fatima, 2018). According to the resource desperation principle of resource conservation theory, in order to get rid of desperation, individuals with exhausted resources will trigger self-defense mechanisms and make offensive and irrational behaviors to promote changes in stressors or promote new resource strategies (Hobfoll et al., 2018). The deviating behavior from the perspective of resource preservation is, actually, the self-defense behavior of an individual to get rid of the stressor in the desperate situation of resources. Empirical data also support the positive correlation between emotional exhaustion and deviant behavior (Mulki et al., 2006; Kong et al., 2020).

In summary, considering the negative correlation between caring and coaching help and emotional exhaustion, and the positive correlation between emotional exhaustion and deviant behavior, we thus hypothesize the following:

H2a: Emotional exhaustion mediates the relationship between caring helping and workplace deviance.

H2b: Emotional exhaustion mediates the relationship between coaching helping and workplace deviance.

H2c: Emotional exhaustion mediates the relationship between substituting helping and workplace deviance.

### The Moderating Roles of Extrinsic Career Goals

Consistent with conservation of resources theory, the resources needed by individuals to cope with stress include personal characteristics, conditions, energy, and material resources. These resources play an important role in obtaining or producing valuable resources (Hobfoll, 2001). Individuals' judgments on resources are subjective. Due to the differences in personal goals, different individuals will get different value judgments when evaluating the same thing (Halbesleben et al., 2014; Hobfoll et al., 2018). When the individual perceives that a certain helping behavior helps to achieve personal goals, the behavior can activate resource gain; on the contrary, the behavior may induce accelerated resource depletion (Hobfoll et al., 2018). Existing studies have shown that pro-social motivation and regulatory focus can affect individuals' value judgments of helping behaviors in the workplace (Koopman et al., 2016; Lanaj et al., 2016; Lin et al., 2017). However, few studies have focused on the impact of employees' career goals. We suspect employees of high extrinsic career goals likely respond more positively to the resource-depleting effects caused by workplace helping.

Career goals represent the career results that employees strive to achieve (Seibert et al., 2013). It is a personal goal for a specific job or a specific attribute in the job, including the pursuit of extrinsic career goals such as obvious success, status, income, influence, and the pursuit of intrinsic career goals, such as new knowledge, new skills, and engaging in challenging, meaningful, and valuable work (Seibert et al., 2013). Different from intrinsic ones, the achievement of extrinsic career goals (i.e., the pursuit of income and promotion) is more closely related to in-role performance (Bergeron et al., 2013), with which workplace helping will compete for a resource (Bergeron, 2007). Scholars have, in the past, viewed these two forms of career goals as theoretically and empirically independent (Seibert et al., 2013). Based on this, we believe that extrinsic career goals are more likely to affect individuals' value judgments of helping behavior in the workplace.

From the perspective of resource conservation theory, we will discuss the fit between the three types of workplaces helping behaviors and extrinsic career goals, and explore the influence of extrinsic career goals on the relationship between workplace helping behaviors and emotional exhaustion. First of all, caring helping requires the helper to invest in emotional resources, which has little impact on the helper's task performance, but the gratitude and good interpersonal relationship cannot be directly converted into visible extrinsic work rewards (such as salaries and career advancements) (Bergeron et al., 2013). For individuals with high extrinsic career goals, caring helping is inconsistent with their personal goals. Secondly, coaching helping also has a small impact on the job, but by sharing knowledge and skills with colleagues, it can show the advantages of the helper in the organization (He et al., 2020a, 2021). According to the perspective of evolutionary psychology, coaching helping is conducive to improving the status and image of the helper in the organization (Salamon and Deutsch, 2006), in line with the goals of individuals with high extrinsic career goals. Finally, substituting helping will cost a lot of resources, have a negative impact on their own work, damage the helper's task performance improvement, and have an adverse effect on the improvement of work income and status. It is at odds with the personal goals of individuals with high extrinsic goals (Bergeron, 2007). In summary, individuals with high extrinsic career goals will regard coaching helping as resource gaining behaviors, while caring and substituting helping as resource depletion behaviors. When individuals with high extrinsic career goals implement coaching helping, it is more conducive to the helpers to obtain resources and alleviate their emotional exhaustion; when they implement caring helping, they are prone to resource loss, which offsets the resource recovery of caring helping. When they implement substituting helping, the depletion effect on resources is more significant, resulting in more serious emotional exhaustion. We thus hypothesize the following:

H3a: Extrinsic career goals moderate the relationship between caring helping and emotional exhaustion. When the extrinsic career goals are lower, the negative correlation between caring helping and emotional exhaustion is more significant.

H3b: Extrinsic career goals moderate the relationship between coaching helping and emotional exhaustion. When the extrinsic career goals are higher, the negative correlation between coaching helping and emotional exhaustion is more significant.

H3c: Extrinsic career goals moderate the relationship between substituting helping and emotional exhaustion. When the extrinsic career goals are higher, the positive correlation between substituting helping and emotional exhaustion is more significant.

In addition, based on the previously discussed assumptions, when the extrinsic career goals are lower, the negative impact of caring helping on emotional exhaustion is more significant, and emotional exhaustion is positively correlated with workplace deviance. We have reason to infer that when employees have low extrinsic career goals, caring helping has a greater impact on the negative indirect effect of workplace deviance through emotional exhaustion. Similarly, since the higher the extrinsic career goals, coaching helping has a more significant negative impact on emotional exhaustion, and emotional exhaustion is positively correlated with workplace deviance, we infer that when employees have high extrinsic career goals, coaching helping has a greater impact on the negative indirect effects of workplace deviance through emotional exhaustion. Because the higher the extrinsic career goals, the more significant the positive impact of substituting helping on emotional exhaustion, and emotional exhaustion is positively correlated with workplace deviance, we assume that when employees have high extrinsic career goals, substitutional helping has a greater positive and indirect effect on workplace deviance through emotional exhaustion.

H4a: Extrinsic career goals moderate the indirect effect between caring helping and workplace deviance *via* emotional exhaustion. When the extrinsic goals are lower, the indirect effect is more significant.

H4b: Extrinsic career goals moderate the indirect effect between coaching helping and workplace deviance *via* emotional exhaustion. When the extrinsic goals are higher, the indirect effect is more significant.

H4c: Extrinsic career goals moderate the indirect effect between substituting helping and workplace deviance *via* emotional exhaustion. When the extrinsic goals are higher, the indirect effect is more significant.

## Method

### Participants and Procedures

Data were collected from full-time employees from eight information technology (IT) companies in China from January to March 2020. We intentionally recruited participants from IT industry because they are mainly engaged in team-based work such as software development, and interpersonal helping is a common occurrence in the organization. Before data collection, all 800 respondents were announced to be assured of their voluntary and confidential participation. We conducted three waves of surveys using a code on the questionnaires to link them. At Time 1, respondents were required to report their levels of caring helping, coaching helping, substituting helping, extrinsic career goals, and control variables (i.e., age, gender, education level, tenure, neuroticism, and agreeableness). At Time 2 (a month after Time 1), the respondents who answered every scale at Time 1 again reported their levels of emotional exhaustion. At Time 3 (2 months after Time 1), the respondents who answered every scale at Time 2 reported their levels of workplace deviance.

About 658 questionnaires of Time 1 were returned at a response rate of 85.25%, 546 of Time 2 and 512 of Time 3. Thus, among the 800 respondents, 512 of them answered every wave of the questionnaire at a full response rate of 64.00% (250 males, 262 females). They are aged 20 to 55 (*M* = 38.64 years, *SD* = 8.97 years), and more than 80% had a university degree or equivalent. Regarding their work contexts, 98.05% of the participants worked for more than 1 year.

The questionnaire process consisted of three time points to meet the needs of the study while reducing the potential for common method bias (Podsakoff et al., 2003). Prior to the study, the participants had voluntarily signed informed consent and were allowed to withdraw at any time. In the first wave of the study, the participants reported on their current extrinsic career goals, agreeableness, neuroticism, demographic information (including gender, age, education level, and tenure), caring, coaching, and substituting helping. In the second wave of the study (a month after the first wave), the participants rated their emotional exhaustion. The third wave of the research (a month after the second wave) focused on measuring the workplace deviance of the participants. Given the secretive nature of workplace deviance and the difficulty of identifying it using direct observation (Fox et al., 2001), we used a self-report questionnaire to measure workplace deviance in our research, again promising the participants the anonymity of this study in order to mitigate concerns of social desirability bias.

### Measures

All English-based scales were translated into Chinese according to Brislin (1970)'s procedures to ensure consistency in meaning with the original. To provide more descriptions and increase the probability of fitting the feeling of the respondents, a 7-point Likert scale was used for all scales, with 1 being “totally disagree” and 7 being “totally agree” (Cox, 1980).

#### Caring, Coaching, and Substituting Helping

Caring, coaching, and substituting helping were measured at Time 1. Based on scales developed by previous research (Williams and Anderson, 1991; Van Dyne et al., 1994; Farh et al., 1997; Podsakoff et al., 1997; Lee and Allen, 2002; Settoon and Mossholder, 2002; McDonald et al., 2018), this study used items analysis, exploratory factor analysis, and confirmatory factor analysis; we developed the scales of caring, coaching, and substituting helping. Results revealed three distinct factors, with all items significantly loading above 0.52 only on their *a priori* factor. Accordingly, we averaged the four items to measure caring helping (a sample item is “I often help colleagues overcome negative affect”; α = 0.85), the six items to measure coaching helping (a sample item is “I share knowledge with colleagues frequently.”; α = 0.89) and the remaining five items to measure substituting helping (a sample item is “I often assist coworkers with heavy workloads even though it is not part of job.”; α = 0.87).

#### Extrinsic Career Goals

Extrinsic career goals were assessed at Time 1 using a 5-item version of Seibert et al. (2013)'s measure (α = 0.74). A sample item is “It is important to me to achieve financial success in my career.”

#### Emotional Exhaustion

We adopted Watkins et al. (2015)'s measure to reflect emotional exhaustion of the participants at Time 2 (α = 0.92). Sample items are “I feel emotionally drained from my work,” “I feel burned out from my work,” and “I feel exhausted when I think about having to face another day on the job.”

#### Workplace Deviance

Workplace deviance was measured at Time 3, selecting 10 items from the version of Bennett and Robinson (2000)'s measure (α = 0.81 for the organizational deviance; α = 0.78 for the interpersonal deviance). Sample items include “Made fun of someone at work” and “Spent too much time fantasizing or daydreaming instead of working.”

#### Control Variables

Consistent with previous research (Yam et al., 2017), we measured age, gender, education level, and tenure at Time 1 to control for their potentially spurious effects. Agreeableness was measured and included as a control variable in the analyses because previous research has demonstrated that they may be related to workplace deviance (Berry et al., 2007). Moreover, because previous research suggests that neuroticism can influence self-reported perception and hence contribute to common method bias (Podsakoff et al., 2003), we measured neuroticism and agreeableness at Time 1 using 12 items each from Costa and McCrae (1992)'s NEO Five-Factor Inventory scale.

### Data Analysis

Firstly, since the same self-report method was adopted, the correlation between variables mentioned above may owe to common method bias (Podsakoff et al., 2003). Harman's single-factor test and controlling for the effects of an unmeasured latent method factor (ULMC) are applied to detect common method bias. Harman's single-factor results indicate the loading on a single factor explains 38.57% of total variance, lower than 50% recommended by Podsakoff et al. (2003). The confirmatory factor analysis (CFA) with the unmeasured latent method factor was conducted to test the potential impact of common method bias. Items were allowed to load on an unmeasured latent construct as a common method variance (CMV) factor in the confirmatory factor analysis. Results showed that, compared to original CFA model fit (χ^2^/df = 2.628, TLI = 0.961, CFI = 0.964, RMSEA = 0.056), the unmeasured latent construct failed to improve CMV model fit (χ^2^/df = 2.476, TLI = 0.965, CFI = 0.970, RMSEA = 0.054) significantly, indicating common method variance is not a pervasive problem in this study.

Secondly, the analyses were conducted with the structural equation modeling (SEM) approach using Amos 23.0. Under the model, caring, coaching, and substituting helping were directly and indirectly (through emotional exhaustion) associated with workplace deviance. The Chi-square likelihood ratio statistic, the Tucker and Lewis Index (TLI), the Comparative Fit Index (CFI), the Root Mean Square Error of Approximation (RMSEA) were used to evaluated the fit of model. According to Carmines and McIver (1981), a smaller value of Chi-square likelihood ratio indicates a better fit of model. TLI and CFI are recommended to be >0.95 (Hu and Bentler, 1998), and RMSEA values lower than 0.08 (Browne and Cudeck, 1993).

Finally, the moderating effects of extrinsic career goals were examined using Model 7 for PROCESS (Hayes, 2013).

## Results

### Statistical Description and Correlation Analysis

Table 1 shows the descriptive statistics and correlations among the variables.

**Table 1:** Descriptive statistics and correlations among all variables.

**Variables**	**1**	**2**	**3**	**4**	**5**	**6**	**7**	**8**	**9**	**10**	**11**	**12**
(1) Gender												
(2) Age	−0.03											
(3) Education	0.02	−0.08										
(4) Tenure	−0.10^*^	0.59^**^	−0.04									
(5) Neuroticism	0.03	−0.13^**^	0.01	−0.20^**^								
(6) Agreeableness	−0.05	−0.03	0.01	−0.07	−0.17^**^							
(7) Caring helping	−0.09^*^	0.01	0.10^*^	0.08	−0.12^**^	0.27^**^						
(8) Coaching helping	−0.07	−0.02	0.06	0.01	−0.07	0.10^*^	0.29^**^					
(9) Substituting helping	−0.02	−0.01	−0.00	0.13^**^	−0.08	0.10^*^	0.39^**^	0.25^**^				
(10) Emotional exhaustion	−0.05	−0.03	−0.08	−0.06	0.14^**^	−0.00	−0.10^*^	−0.38^**^	0.31^**^			
(11) Extrinsic goal career	0.11^*^	0.03	0.03	0.03	0.06	−0.05	−0.10^*^	−0.62^**^	−0.03	0.45^**^		
(12) Workplace deviance	0.05	−0.01	−0.06	0.03	0.06	−0.02	−0.12^**^	−0.60^**^	0.17^**^	0.69^**^	0.61^**^	
Mean	1.51	38.64	4.97	2.53	2.79	5.71	5.57	5.11	5.39	2.90	2.19	1.99
SD	0.50	8.97	0.56	0.70	1.40	0.92	1.00	1.81	1.59	1.61	1.21	1.72

*N = 512. Gender: 1 = male and 2 = female; Education: 1 = primary school, 2 = junior high school, 3 = high school, 4 = college degree, 5 = bachelor's degree, 6 = master's degree, 7 = doctor's degree; Tenure: 1 = <1 year, 2 = 1–5 years, 3 = 6–10 years, 4 = more than 10 years*.

* *p < 0.05*,

** *p < 0.01 (two-tailed test)*.

### Confirmatory Factor Analysis

Before hypotheses testing, we had first conducted confirmatory factor analysis (CFA) to examine whether the measured constructs had discriminant validity. As shown in Table 2, CFA results indicated that, compared to other alternative models, the hypothesized 6-factor model fit the data better: $\chi_{\left(578\right)}^{2}$ = 1549.048, χ^2^/df = 2.628, TLI = 0.959, CFI = 0.963, RMSEA = 0.057. Thus, the distinctiveness of the focal constructs was supported.

**Table 2:** Results of the confirmatory factor analysis for the main variables.

**Factor models**	**χ^2^**	***df***	**χ^2^/df**	**CFI**	**TLI**	**RMSEA**
Single-factor model: CAH+COH+SUH+EE+ECG+WD	11657.610	592	19.692	0.576	0.549	0.191
Two-factor model 1: CAH+COH and SUH+EE+ECG+WD	8489.713	591	14.365	0.697	0.677	0.162
Two-factor model 2: CAH+COH+SUH and EE+ECG+WD	8509.341	591	14.398	0.697	0.677	0.162
Three-factor model 1:CAH and COH and SUH+EE+ECG+WD	6857.587	589	11.643	0.760	0.743	0.144
Three-factor model 2:CAH+COH and SUH and EE+ECG+WD	5303.754	589	9.005	0.819	0.807	0.125
Four-factor model 1:CAH and COH and SUH and EE+ECG+WD	3618.963	586	6.176	0.884	0.875	0.101
Four-factor model 2:CAH+ COH and SUH and EE and ECG+WD	4037.717	586	6.890	0.868	0.858	0.107
Five-factor model 1:CAH and COH and SUH and EE and ECG+WD	2346.294	582	4.031	0.932	0.927	0.077
Five-factor model 2:CAH+COH and SUH and EE and ECG and WD	3242.753	582	5.572	0.898	0.890	0.095
Five-factor model 3:CAH and COH and SUH and EE+ECG and WD	2874.592	582	4.939	0.912	0.905	0.088
Six-factor model	1549.048	578	2.680	0.963	0.959	0.057

*CAH, caring helping; COH, coaching helping; SUH, substituting helping; EE, emotional exhaustion; ECG, extrinsic career goals; and WD, workplace deviance*.

### Hypotheses Testing

According to the results of the hierarchical multiple regression analysis in Table 3, caring and coaching helping were both negatively related to workplace deviance (β_1_ = −0.111, *p* < 0.1; β_2_ = −0.631, *p* < 0.01; Model 6), while substituting helping was positively related to workplace deviance (β = 0.384, *p* < 0.01; Model 6). Thus, Hypotheses 1a, 1b, and 1c were supported.

**Table 3:** Results of hierarchical regression analyses.

**Variables**	**Emotional exhaustion**					**Workplace deviance**	
	**Model 1**	**Model 2**	**Model 3**	**Model 4**	**Model 5**	**Model 6**	**Model 7**
**Control variables**							
Gender	−0.287^*^	−0.350^**^	−0.315^**^	−0.366^***^	−0.393^***^	0.030	0.182
Age	0.125	0.121	0.129	0.142	0.115	−0.072	−0.139
Education	−0.094	−0.151	−0.123	−0.093	−0.083	−0.060	−0.010
Tenure	−0.259^*^	−0.278^**^	−0.272^**^	−0.272^**^	−0.298^**^	0.053	0.192^*^
Neuroticism	0.144^***^	0.132^**^	0.134^***^	0.134^***^	0.128^**^	0.050	−0.027
Agreeableness	0.079	0.078	0.037	0.075	0.068	0.067	0.025
**Independent variables**							
Caring helping	−0.248^***^	−0.272^***^	−0.340^***^	0.403^***^	−0.336^***^	−0.111^†^	0.022
Coaching helping	−0.402^***^	−0.230^***^	−0.216^***^	−0.146^**^	−0.140^**^	−0.631^***^	−0.416^***^
Substituting helping	0.506^***^	0.471^***^	0.474^***^	−0.276^***^	0.459^***^	0.384^***^	0.115^**^
**Moderator**							
Extrinsic career goals		0.389^***^	0.418^***^	0.212^**^	0.469^***^		
**Interaction**							
Caring helping*Extrinsic career goals			0.188^***^				
Coaching helping*Extrinsic career goals				−0.128^***^			
Substituting*Extrinsic career goals					0.184^***^		
**Mediator**							
Emotional exhaustion							0.533^***^
**Constant**	4.004^***^	3.068^***^	2.333^**^	2.638^**^	6.227^***^	3.537^***^	1.401^*^
**R-sq**	0.371	0.420	0.443	0.442	0.459	0.469	0.625

*N = 512*,

† *p < 0.1*,

* *p < 0.05*,

** *p < 0.01*,

*** *p < 0.001*.

Next, we tested the mediating effects of emotional exhaustion proposed in Hypotheses 2a, 2b, and 2c. Standardized mediation analysis results presented in Table 4 are based on 5,000 bootstrap replications using the bias-corrected percentile bootstrap method. Table 4 shows that the indirect effect of caring helping *via* emotional exhaustion on workplace deviance was −0.132 [95% CI = (−0.228, −0.036)]; the indirect effect of coaching helping *via* emotional exhaustion on workplace deviance was −0.215 [95% CI = (−0.268, −0.160)]; the indirect effect of substituting helping *via* emotional exhaustion on workplace deviance was 0.270 [95% CI = (0.201, 0.336)]. Thus, Hypotheses 2a, 2b, and 2c were supported.

**Table 4:** Standardized mediation analysis results.

**Model paths**	**Estimate**	**SE**	**95% LLCI**	**95% ULCI**	**90% LLCI**	**90% ULCI**
**Total effect**						
Caring helping → Workplace deviance	−0.111	0.065	−0.237	0.087	−0.217	−0.004
Coaching helping → Workplace deviance	−0.631	0.033	−0.695	−0.567	−0.685	−0.577
Substituting helping → Workplace deviance	0.384	0.039	0.307	0.461	0.320	0.449
**Direct effect**						
Caring helping → Workplace deviance	0.022	0.055	−0.087	0.696	−0.069	0.112
Coaching helping → Workplace deviance	−0.416	0.031	−0.478	−0.355	−0.468	−0.365
Substituting helping → Workplace deviance	0.115	0.038	0.040	0.189	0.052	0.177
**Indirect effect**						
Caring helping → Emotional exhaustion → Workplace deviance	−0.132	0.049	−0.228	−0.036	−0.214	−0.050
Coaching helping → Emotional exhaustion → Workplace deviance	−0.215	0.028	−0.268	−0.160	−0.262	−0.170
Substituting helping → Emotional exhaustion → Workplace deviance	0.270	0.035	0.201	0.336	0.214	0.326

**Figure 2:**
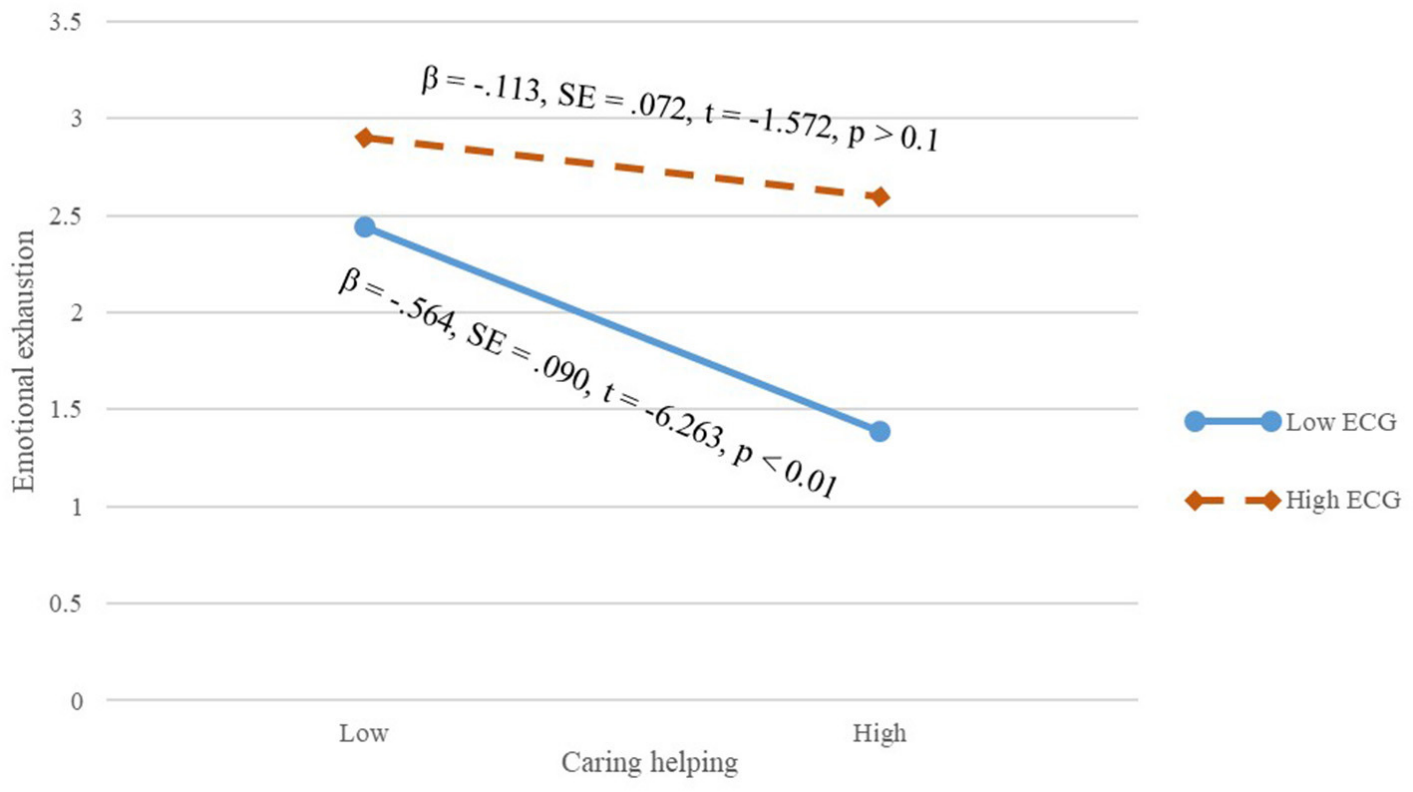
Interactive effect of extrinsic career goals on the relationship between caring helping and employees' emotional exhaustion.

**Figure 3:**
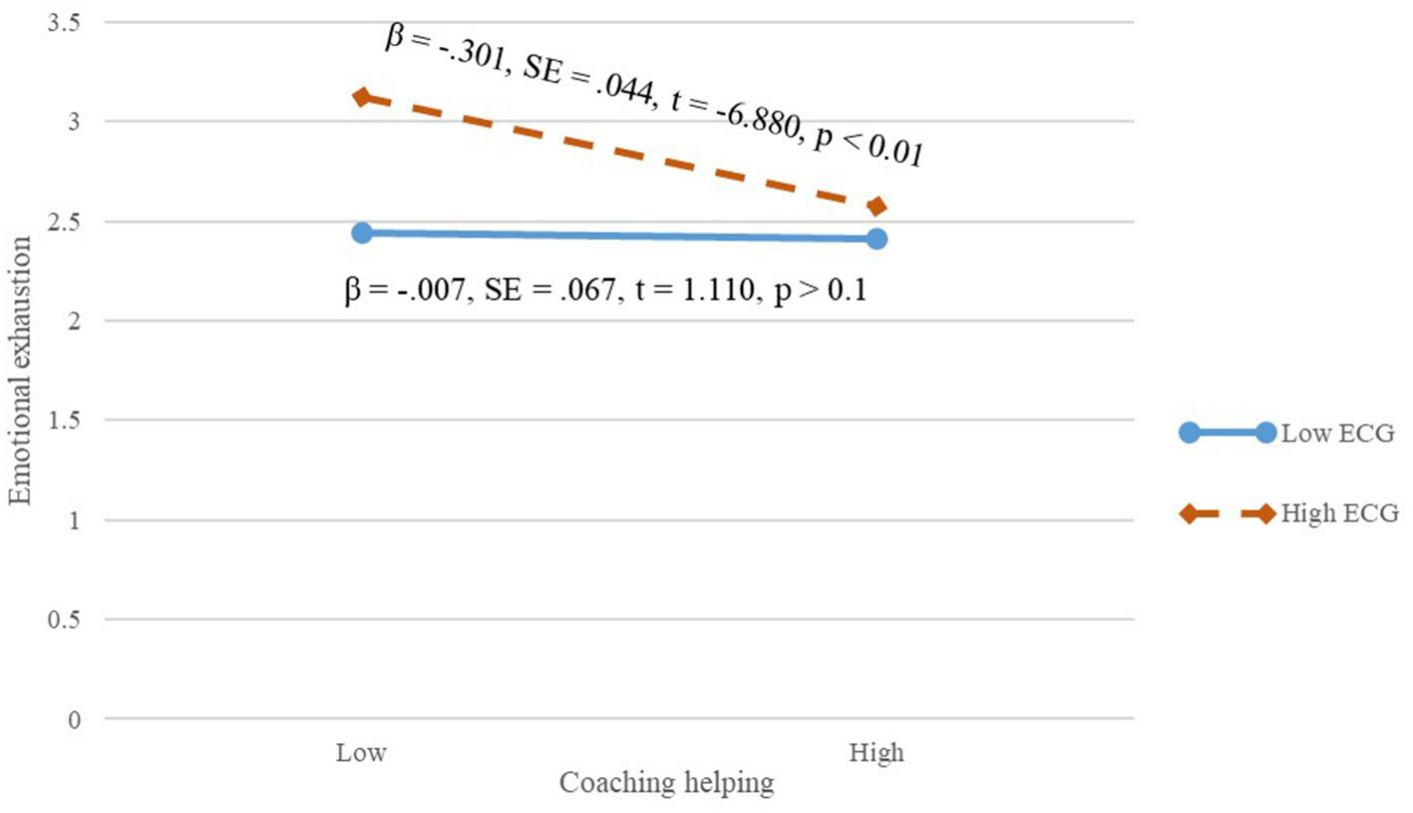
Interactive effect of extrinsic career goals on the relationship between coaching helping and employees' emotional exhaustion.

**Figure 4:**
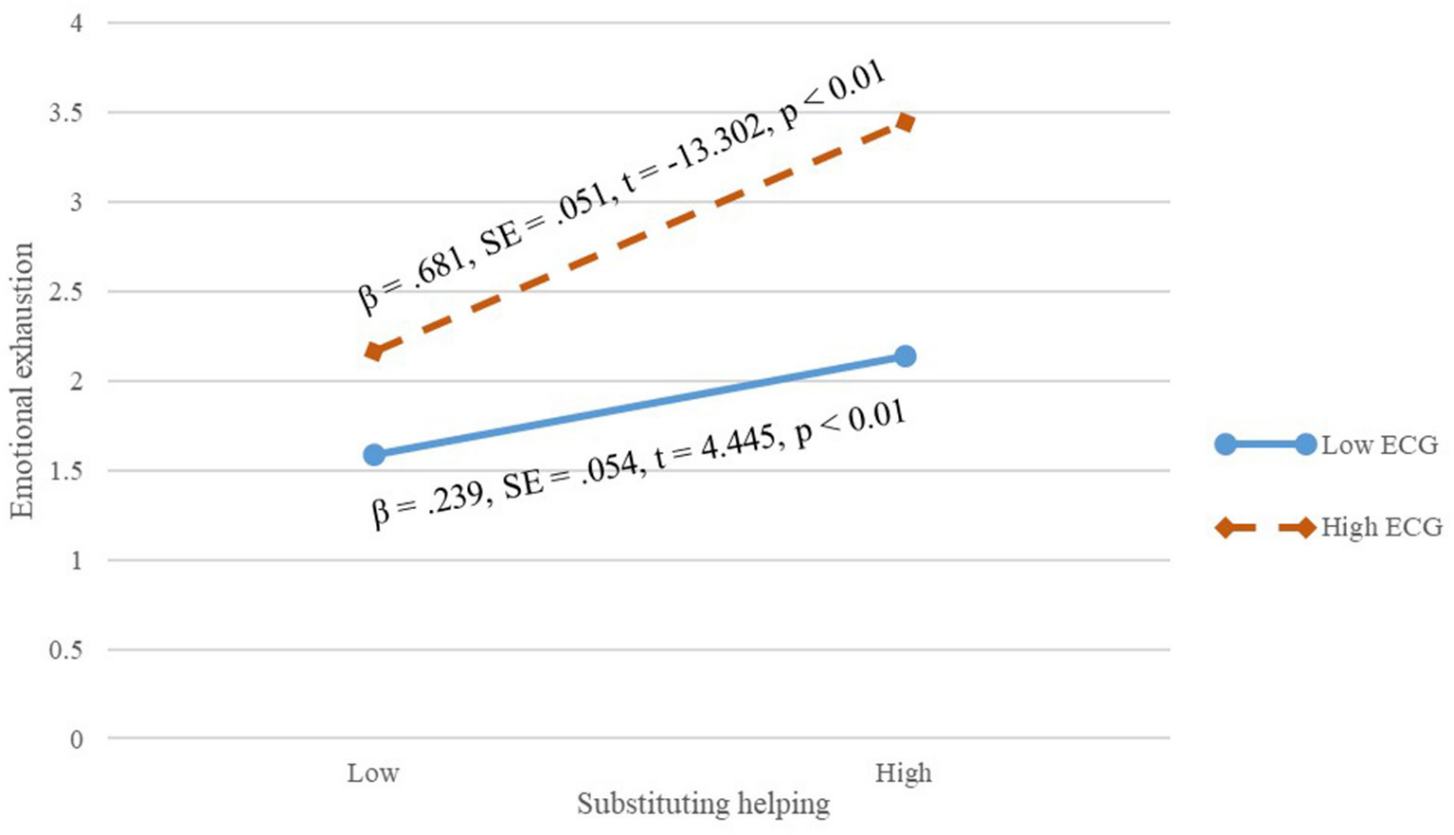
Interactive effect of extrinsic career goals on the relationship between substituting helping and employees' emotional exhaustion.

In addition, we then tested the moderating effects of extrinsic career goals. Based on the results of the hierarchical multiple regression analysis in Table 3, the interaction term of caring help and extrinsic career goals was significantly and positively associated with emotional exhaustion (β = 0.188, *p* < 0.01; Model 3). The interaction term of coaching help and extrinsic career goals was significantly and positively associated with emotional exhaustion (β = −0.128, *p* < 0.01; Model 4). The interaction term of substituting help and extrinsic career goals was significantly and positively associated with emotional exhaustion (β = 0.184, *p* < 0.01; Model 5). As shown in Figure 2, when extrinsic career goals were low, caring helping was more negatively related to workplace deviance (β = −0.564, *SE* = 0.090, *t* = −0.263, *p* < 0.01) than when extrinsic career goals were high, and, thus, Hypothesis 3a was supported. As shown in Figure 3, when extrinsic career goals were high, coaching helping was more negatively related to workplace deviance (β = −0.301, *SE* = 0.044, *t* = −0.880, *p* < 0.01) than when extrinsic career goals were low, and, thus, Hypothesis 3b was supported. As shown in Figure 4, when extrinsic career goals were high, substituting helping was more positively related to workplace deviance (β = 0.681, *SE* = 0.051, *t* = −13.302, *p* < 0.01) than when extrinsic career goals were low, and, thus, Hypothesis 3c was supported.

The bias-corrected percentile bootstrap method with SPSS further indicated that the indirect effects of three types of helping on workplace deviance through emotional exhaustion were moderated by extrinsic career goals. Table 5 shows that the indirect effect for caring helping → emotional exhaustion → workplace deviance was stronger and significant for employees in low extrinsic career goals [β = −0.301, 95% IC = (−0.451, −0.161)], but was not significant for those in high extrinsic career goals [β = −0.060, 95% IC = (0.132, 0.031)]; the indirect effect for coaching helping → emotional exhaustion → workplace deviance was stronger and significant for employees in high extrinsic career goals [β = −0.160, 95% IC = (−0.217, −0.113)], but was not significant for those in low extrinsic career goals [β = 0.004, 95% IC = (0.057, 0.067)]; the indirect effect for substituting helping → emotional exhaustion → workplace deviance was stronger and significant for employees in high extrinsic career goals [β = 0.363, 95% IC = (0.285, 0.452)], but was weaker but significant for those in low extrinsic career goals [β = 0.127, 95% IC = (0.081, 0.175)]. Finally, the index of moderated mediation indicated that the moderated mediation effects of extrinsic career goals were significant, thereby supporting Hypotheses 4a, 4b, and 4c.

**Table 5:** Moderated mediation results.

**Moderator variable**	**Estimate**	**SE**	**95% LLCI**	**95% ULCI**
**Caring helping → Emotional exhaustion → Workplace deviance**				
Extrinsic career goals low	−0.301	0.075	−0.451	−0.161
Extrinsic career goals mean	−0.181	0.050	−0.278	−0.081
Extrinsic career goals high	−0.060	0.042	−0.132	0.031
IMM	0.101	0.028	0.054	0.163
**Coaching helping → Emotional exhaustion → Workplace deviance**				
Extrinsic career goals low	0.004	0.032	−0.057	0.067
Extrinsic career goals mean	−0.078	0.023	−0.125	−0.035
Extrinsic career goals high	−0.160	0.027	−0.217	−0.113
IMM	−0.069	0.015	−0.101	−0.041
**Substituting helping → Emotional exhaustion → Workplace deviance**				
Extrinsic career goals low	0.127	0.024	0.081	0.175
Extrinsic career goals mean	0.245	0.028	0.191	0.301
Extrinsic career goals high	0.363	0.042	0.285	0.452
IMM	0.098	0.016	0.069	0.134

*IMM, index of moderated mediation*.

## Discussion

Although prior studies have noted the importance of workplace helping, little is known about how styles of helpers' helping influence their subsequent behaviors (e.g., workplace deviance). The main purpose of our research was to integrate a clear theoretical framework to understand influences of three types of workplaces helping on helpers' subsequent deviant behaviors. Our conceptual framework was proposed from the perspective of the conservation of resources theory to explore the mechanism of the effect and boundary conditions of caring, coaching, and substituting helping on subsequent workplace deviance and supported by empirical pieces of evidence from China using a three-wave time-lagged design. We found that caring and coaching helping were both negatively related to emotional exhaustion, while substituting helping was positively related to emotional exhaustion, which was negatively related to workplace deviance. Taken together, the findings suggest that, on one hand, caring and coaching helping weakens helpers' subsequent deviant behaviors by reducing their emotional exhaustion. On the other hand, by inducing helpers' emotional exhaustion, substituting helping increases the potential to damage their organization or colleagues. The outcomes of the present study imply that caring and coaching helping should be good for helpers, while substituting helping would hurt them. The results further indicated that extrinsic career goals played a moderating role between three types of workplaces helping and workplace deviance, and moderated the mediating effect of emotional exhaustion. This finding also points to the fact that helpers of high extrinsic career goals tend to feel more emotionally exhausted after doing caring and substituting helping. We next discuss the theoretical and practical implications of these findings.

### Theoretical Implications

A first contribution this study makes to the workplace helping literature is found in the development of the caring, coaching, and substituting helping constructs. Compared with situations and contents of helping (Spitzmuller and Van Dyne, 2013; Bamberger et al., 2017), relatively little research attention has been focused on the styles of helping in the field of helping types. Based on the effort a helper makes to help coworkers, we have divided workplace helping into three types: caring helping, coaching helping, and substituting helping. From the perspective of helping styles of helpers, we developed a board measure of workplace helping that we found to be related to helpers' psychological resources and their subsequent behaviors. Our findings showed, contrary to substituting helping, caring and coaching helping were more negatively related to emotional exhaustion, and the helpers of these two styles were less likely to commit subsequent deviant behaviors. Thus, the distinction between helping styles of helpers proves to be important in explaining the influences of helping on helpers.

A second contribution this study makes is to improve our knowledge of how and when a helpful employee deviates. Workplace deviance of a helper has been considered to be caused by external factors (Yam et al., 2017; Koopman et al., 2020). Our findings revealed that choosing resource-exhausting ways (i.e., substituting helping) to help co-workers can also lead to deviant behaviors. For helpers, helping styles have an impact on their resource allocation and subsequently influence the coping strategies under pressure. Substituting helping can put the helper in a desperate resource situation, and the helper has to resort to aggressive behavior (i.e., taking advantage of the organization or colleagues) to get out of the situation (Hobfoll et al., 2018). The theoretical model validates our theoretical perspective.

Finally, our research has contributed to apply conservation of resources theory to career goals. Researchers have paid little attention to extrinsic career goals in the field of the conservation of resources theory; however, our results suggest that extrinsic career goals have important implications for the way in which workplace helping is evaluated by helpers. Employees with high extrinsic career goals view helping behaviors that are consistent with their goals as resource acquisition behaviors and *vice versa* as resource depletion behaviors (Hobfoll et al., 2018). Our work, therefore, serves as a catalyst for further examinations of career goals as a moderator in the literature based on the conservation of resources theory.

### Managerial Implications

Workplace helping is essential to organizations, and managers welcome the increasing amount of helping. However, some type of helping is resource depleting and ultimately induces in future deviance in the workplace. Based on the results of this study, the following managerial implications have been proposed:

First, organizations should focus on mentoring helpful employees to reduce emotional exhaustion by choosing appropriate ways to help in order to prevent subsequent deviant behaviors of them. For example, tips for helping colleagues at work should be provided. As such, employees would know when and how to support colleagues at work in the right way. Managers need to be aware that allowing employees to engage in high levels of substituting helping can inadvertently hurt them, who probably harm the organization and other employees in turn. Organizations should identify excessive substituting helping in a timely manner and compensate helpers with resources or replace them with others. For instance, leaders schedule meetings with subordinates to communicate work progress and encourage subordinates to share their concerns about resources.

Second, different indirect effects of three types of workplaces helping on workplace deviance *via* emotional exhaustion remind helpers of appropriately using helping strategies with discretion. For example, when employees perceive the lack of resources, it may be wiser for them to provide caring and coaching helping than to provide substituting helping for others because substituting helping could further leave them emotionally drained and exhausted. Organizational norms of workplace helping could both limit the excessive substituting helping and encourage caring and coaching helping.

Finally, organizations that value workplace helping may benefit from selecting on the interaction of career goals and types of helping that make employees less vulnerable to the resource-depleting effects of OCB, such as extrinsic career goals. For example, managers should not encourage employees with high extrinsic career goals to help colleagues by caring and substituting helping. Organizations, therefore, ought to recognize that individual differences in career goals have significant impact on the evaluation of resource-related behaviors of helpers. Furthermore, Human Resources Development Department could implement policies and procedures that clarify each employee's career goal orientation (Greco and Kraimer, 2020).

### Limitations and Directions for Future Research

Despite these theoretical and practical implications, this study is not without limitations. The first limitation is that, although some precautions have been taken to limit common method bias, reasonable concerns still remain for using the self-report strategy for data collection. Time-lagged design was used to separate the measurement of independent, mediating, and dependent variables, reducing the influences of the participants' transient moods and response styles (Rindfleisch et al., 2008). The participants' personality traits (e.g., agreeableness and neuroticism) were controlled to limit the effect of the participants' response tendencies on common method bias (Podsakoff et al., 2003). Nevertheless, future research could improve our design by bringing in observers to rate focal variables or an experimental replication of our findings.

A second limitation of our work is that, although the mediating and moderating mechanisms for the effects of different types of workplaces helping on deviance, other mechanisms may also be in existence to influence these effects. For example, in terms of moderators, anticipating gratitude from a recipient was regarded as one of the ways in which the psychological resources of the helper are restored (Lee et al., 2019). The interaction between the helper and the recipient may moderate the resource acquisition and depletion processes in helping events.

Finally, research data come exclusively from employees working in China. Chinese culture values harmony in interpersonal relationships, and Chinese employees are aware of the fact that organizations expect them to lend a helpful hand to colleagues in trouble (i.e., compulsory citizenship behavior; He et al., 2020b). In other words, workplace helping measured in Chinese cultural context may be overestimated (Lin et al., 2020). Therefore, it remains to be further empirically tested whether the findings of this study remain valid for companies in other cultural contexts.

In terms of future research directions, this study only explored workplace helping of coworkers at the same hierarchical organizational level, and future research could build on our work by extending to cross-level helping behaviors between leaders and their subordinates. Based on the social cognitive theory, individuals can gain vicarious experiences by observing the success of others, enhancing their self-efficacy (Wood and Bandura, 1989). Similarly, employees gain a greater sense of self-efficacy by closely observing their leaders' helping behaviors and successfully adopting helping behaviors toward their colleagues (Zhang et al., 2020). Moreover, it would be interesting to investigate cross-level workplace helping because there could be many differences in the types and impacts of helping due to the status gap between leaders and their subordinates (Harari et al., 2021).

Another needed direction for future research is to test our theoretical model in another cultural context. Our theoretical model is tested in Chinese culture, which is described as more collectivist (Lin et al., 2020). Workplace helping in organizations of American culture, which is described as more individualistic, may be different because helpers may be more reciprocally motivated (Spitzmuller and Van Dyne, 2013). Future research that compares workplace helping under different cultures would, therefore, be of great value.

Finally, there may be value to use different theoretical lens. Our research provides theoretical explanations for workplace helping and deviance from the perspective of conservation of a resource. Scholars have also drawn from moral licensing theory to suggest the relationship between OCB and deviance *via* psychological entitlement (Yam et al., 2017). Employees may feel psychologically entitled or empowered in varying degrees due to different degrees of effort they make to help coworkers (Yam et al., 2017; Ali et al., 2020).

## Conclusion

Too much engagement in helping can have negative results, but these results are not only related to the amount and frequency of the act itself. A resource-depleting helping (i.e., substituting helping) can also lead to a bad workplace experience for the helpers. Based on the conservation of resources theory, this article extends previous research by proposing a model to examine how and when different types of helping affected helpers' subsequent deviance in the workplace through emotional exhaustion. Our study findings highlight the need to consider the interplay between helping types and individual goals in the process of encouraging workplace helping.

## Data Availability Statement

The raw data supporting the conclusions of this article will be made available by the authors, without undue reservation.

## Ethics Statement

Written informed consent was obtained from the individual(s) for the publication of any potentially identifiable images or data included in this article.

## Author Contributions

HZ designed the research and completed the manuscript. CL and XLa designed the research with HZ and proposed the discussion. XLi revised and checked the whole manuscript in the revision process. All the authors contributed to the article and approved the submitted version.

## Funding

This research project was supported by the National Natural Science Foundation of China (Grant No. 71974059).

## Conflict of Interest

The authors declare that the research was conducted in the absence of any commercial or financial relationships that could be construed as a potential conflict of interest.

## Publisher's Note

All claims expressed in this article are solely those of the authors and do not necessarily represent those of their affiliated organizations, or those of the publisher, the editors and the reviewers. Any product that may be evaluated in this article, or claim that may be made by its manufacturer, is not guaranteed or endorsed by the publisher.
